# Applications of the Soil, Plant and Rumen Microbiomes in Pastoral Agriculture

**DOI:** 10.3389/fnut.2019.00107

**Published:** 2019-07-16

**Authors:** Graeme T. Attwood, Steve A. Wakelin, Sinead C. Leahy, Suzanne Rowe, Shannon Clarke, David F. Chapman, Richard Muirhead, Jeanne M. E. Jacobs

**Affiliations:** ^1^Animal Science, AgResearch, Palmerston North, New Zealand; ^2^Forage Science, AgResearch, Lincoln, New Zealand; ^3^Animal Science, AgResearch, Invermay, New Zealand; ^4^DairyNZ, Lincoln, New Zealand; ^5^Farm Systems and Environment, AgResearch, Invermay, New Zealand

**Keywords:** genomics, metagenomics, pasture, ecosystems, food, soil

## Abstract

The production of dairy, meat, and fiber by ruminant animals relies on the biological processes occurring in soils, forage plants, and the animals' rumens. Each of these components has an associated microbiome, and these have traditionally been viewed as distinct ecosystems. However, these microbiomes operate under similar ecological principles and are connected via water, energy flows, and the carbon and nitrogen nutrient cycles. Here, we summarize the microbiome research that has been done in each of these three environments (soils, forage plants, animals' rumen) and investigate what additional benefits may be possible through understanding the interactions between the various microbiomes. The challenge for future research is to enhance microbiome function by appropriate matching of plant and animal genotypes with the environment to improve the output and environmental sustainability of pastoral agriculture.

## Introduction

The arrival of the next generation sequencing (NGS) era has opened up new opportunities for understanding biological processes and implementing new strategies for improving these processes and monitoring the environmental impacts of agriculture (1–7). This has enabled the development of tools to implement genomic selection (GS) particularly in the livestock industry, and provide genome wide association studies (GWAS) to further elucidate genomic regions of importance in production traits. Genome assemblies, together with re-sequencing, have helped to establish SNP arrays for assessing genetic variation within and between genomes of individuals. This has become well-established for diverse breeds and species from around the globe in animal [e.g., (8–13)], and plant species [e.g., (14–18)]. In addition, genomic selection methods (19–29) used in conjunction with imputation strategies (30–33) that utilize various SNP densities in a cost effective manner, encourage the uptake of GEBVs by the breeding industry with the view to increase the rate of genetic gain in both animals (22) and forage plants (34). Furthermore, utilization of genotypes that are imputed to a whole genome sequence equivalent level for use in GWAS and GS are now a reality (35–39). Continued reductions in DNA sequencing costs together with an improvement in longer read technology has generated more refined genome assemblies that are being annotated at the functional level via assays designed to establish chromatin architecture, accessibility, modification and subsequent transcription and translation profiles (40–45). The human and mouse ENCODE projects (46–49) have paved the way for the international consortium FAANG [Functional Annotation of the Animal Genome; (50, 51)], which aims to identify all the functional elements in animal genomes. A significant challenge in the post-genomic era is connecting genotype to quantitative phenotype in basic and applied biology, represented as the genome to phenome challenge. Understanding the genotype to phenotype link is not only important from a genomic selection perspective but also assists in improving the fundamental understanding of the biology of the system.

The use of genomic tools in livestock research has had a substantial effect on genetic gain for the industry (22). Similar technologies are now being developed and implemented in forage species (34, 52). However, the animal and plant genomes constitute only two components of the “pasture to plate ecosystem.” They exist and function in association with their own microbiomes, and the microbiome of the soil on which pastures are grown and animals graze. The Human Microbiome Project has lead the way in characterizing the contributions that microbiomes make to host phenotypes, with an ever-increasing list of human attributes which are influenced by microbial activities [e.g., (53–69)]. Subsequently, NGS technology has advanced research in the characterization and understanding of the microbiome of the rumen of grazing animals (70), as well as the microbiome of forage plants and soils. Microbiome characterization initially involved sequencing of marker genes within microbial communities mainly targeting rRNA gene sequences but has grown to include deep metagenomic and metatranscriptomic sequencing. This has allowed global characterization of both culturable and unculturable microbial species within an environment coupled with quantification of their gene expression which enables functional profiling. In addition to understanding the contribution microbiomes make to production in grazing animals, genomic techniques can also be extended to monitoring sources of food or water contamination, thereby having important potential impacts for human health.

The integration of genomic information from host organisms with their microbiomes and with other environmental parameters of the target ecosystem has become an important challenge to research projects seeking to enhance agricultural output while reducing its environmental footprint. These host × microbiome × environment relationships in agricultural production systems involve extremely broad and complex interactions along the soil-plant and animal continuum, and its investigation needs to be divided into more specific research questions to enable detailed dissection and analysis. In this context, an “ecological genomics” approach is appropriate, whereby the microbiomes associated with soil, plants and animals are recognized as an integral part of an interconnected system that influence the functions of their hosts and thereby contribute significantly to productive processes in the pastoral sector (71). The key features of, and interactions between the soil-plant-animal microbiomes need to be identified so that their contributions to these agricultural processes and their impacts on the environment can be quantified.

In this paper, we consider the recent developments in genomics that provide new tools to understand the microbiome along the soil-plant-animal continuum within the pastoral production system. We summarize how these tools provide more precision in the identification and quantification of the structure of the microbial communities and how the emerging tools in metagenomics can be applied. Within the soil-plant-animal continuum we look at the animal and farm management opportunities arising from advanced understanding of microbial diversity and ecosystem function and how that can be used to improve soil processes, forage growth and pasture utilization and help withstand the challenges of diseases and climate change. These opportunities are summarized via three case studies involving: the microbiomes of the soil, the pasture, and the rumen of grazing animals. The potential for interdependencies, interplay and interactions between the microbiomes of the ecosystems along this continuum are considered along with other downstream impacts on ecosystems associated with water runoff. We finally propose how an “ecological genomics” approach can contribute to improved understanding of these microbiomes to improve the performance of the pastoral sector.

## Case Study 1: Soil Microbiome

The biology of soils has long been recognized as being central to the productive capacity of natural and managed ecosystems (72, 73). While we cannot directly observe much of the soil microbiome, its function shapes the world around us. Soils microbiomes are highly diverse ecosystems, comprising complex assemblages of bacteria, archaea, and eukaryotic taxa, and are considered the most genetically diverse ecosystems on earth (74). Estimates of the total of life in soil vary widely; bacterial species alone, are present in the order of thousands to tens-of-thousands of species (inferred from 16S rRNA gene phylotypes) per gram of agricultural soil (74–76). Soils provide a reservoir of microbial species that may either support or inhibit the growth of plants and animals directly; as beneficial symbionts or as pathogens, respectively, or indirectly via actions which affect the biological availability of nutrients and toxins (77). Furthermore, functions supported by the soil microbiome provide a range of enabling and provisioning ecosystem services that support the natural environment, including interaction between above- and below-ground terrestrial biomes, aquatic ecosystems (rivers, lakes, groundwater), and the earth's atmosphere [e.g., (78)].

New soil management approaches are aimed at opportunities based on the understanding of soil microbiomes for improved processes and lowered environmental impact (79, 80). These management strategies increasingly use ecological genomics approaches (81) where soil is treated as an ecosystem hosting a rich diversity of species which harbor diverse “functional” genetic elements (e.g., genes conferring antibiotic resistance or nitrogen fixation). Assessing these at an ecosystem level is technologically challenging and requires the development of new bioinformatic and statistical tools for ecological analysis [e.g., (82, 83)]. Most importantly, an ecological genomics approach necessitates a shift in conceptual thinking from the organism or gene using ecosystem property/function interactions, to embracing the complexity of interactions among organisms, their genetic elements, and the biotic and abiotic factors that are expressed collectively to deliver ecosystem processes (84, 85). Ecological genomics offers a key opportunity to further advance the understanding of soil ecology and function and thereby help unravel the complexity of their ecosystems across spatial and temporal scales (86–88).

The application of molecular-based tools has become essential to characterize and understand soil ecosystems, because the soil is hyper-diverse and therefore genetically complex. A single gram of soil is estimated to contain up to 1,000 Gbp of metagenome DNA (80, 89) and current NGS platforms can only provide partial coverage of the metagenomic DNA in a soil sample. To date, most soil metagenome research has relied on the characterization of specific elements within the metagenome. Examples include the use of meta-barcoded primers to assess community composition [e.g., (90)], application of NGS or high-density environmental microarrays to determine functional status/composition of the community [e.g., (91, 92)], or functional screening of libraries of cloned DNA fragments for novel enzymes and bioactive compounds (93).

A good example illustrating metagenomics applications to study the soil microbiome involves soils suppressive to soil-borne plant diseases. These are defined as those in which the activity of the resident soil microbiota reduces the occurrence or severity of plant disease caused by soil-borne pathogens (94). Examples of such disease reduction in soils include suppression in wheat of take-all (*Gaeumannomyces graminis* var. *tritici*) and *Rhizoctonia* bare patch (*Rhizoctonia solani* AG-8) diseases, and the role *Streptomyces* spp. in the plant rhizosphere and endosphere play in promoting plant growth and the induction of resistance via antibiotic production and competitive exclusion (95). Given the high cost of soil-borne disease on agricultural production [e.g., estimated costs of 28–50% of pasture production in New Zealand; (96, 97)] and the lack of practicable and economic control options, the development of disease suppression in soil microbial communities represents an important soil service that serves to maintain agricultural activity and the food and fiber it produces (86). Disease suppression has been observed in a number of soils, with different disease-host interactions, and can develop naturally over time (94, 98). In instances where disease suppression develops, it is underpinned by alteration in the soil microbial community structure toward a greater number of disease suppressive taxa, or expression of potential (latent) disease suppressive activity (86, 99). Not surprisingly, the development of disease suppression in soils is highly desirable, and there have been considerable efforts to understand how this can be facilitated through changes in system management [e.g., via fertilizer use and plant residue management; or via reduced tillage and crop rotation; (94, 100)]. However, the characterization of the community and functions associated with general disease suppression has been very difficult, particularly as they potentially represent a small fraction of the total microbial diversity in soils (101). Furthermore, in the case of “general” disease suppression (102) underpinned by phylogenetically diverse consortia of microbiota, functions such as lytic enzyme production, antibiotic secretion, and elicitation of plant defense mechanisms, may be collectively responsible (94).

Advances in understanding changes within the soil community during development of disease suppression are being supported through application of ecosystem genomic tools [see Dignam et al. (86) review]. These include the application of high density oligonucleotide microarrays (88), tag-based NGS (103), and shotgun metagenomics (104, 105). In each case, phylogenetically diverse microbial consortia were associated with disease suppression. Resolving these taxa against the rich background of soil microbial diversity would not have been possible without an ecological genomics approach.

More recently, the focus on assessment of soil-borne disease suppression has been extended from approaches focused on identifying the taxa responsible, toward assessing soil ecosystems based on functional genes. This has followed recognition that, in many instances, a phylogenetic description of a microorganism can be a poor reflection of the metabolic (functional) ability outside of its base metabolism. This is particularly important where functions, such as antibiotic production, antibiotic resistance, host compatibility, and virulence, are borne on mobile/transferrable genetic elements such as plasmids (106). The acquisition or loss of a plasmid can change the biology and wider ecology of individuals of the same species in the soil. In these cases, the identification of a species only indicates the presence of a “potential host” that may or may not harbor the functional genes of interest [e.g., (107)]. As such, the detection of multiple functional genes associated with disease suppression is likely to provide a richer understanding of the ecosystem potential for this important ecosystem function (108). To achieve this, technology platforms such as functional environmental microarrays (91) are being constantly updated to include information on genes either directly or putatively associated with disease suppression. These include many antibiotic production genes, such as *phz*F and *phz*A (phenazine), *bac*A (bacilysin), *pab*A (chloramphenicol), *phl*D (DAPG), *lgr*D (gramicidin), *lmb*A, (lincomycin), *prn*D (pyrolnitrin), *str*R (streptomycin), *spa*R (subtilin), and *pcb*C (β-lactam) genes, alongside sub-sets of existing gene probes for detection of lytic enzyme production, e.g., *hcn*B (cyanide formation) (109). By assessing the abundance and distribution of these genes in soil environmental DNA (eDNA) samples, the functional ecology of disease suppressive communities may be determined. Impacts of farm management practices on disease suppression can then be interpreted through the lens of functional changes in the soil biology. Over time, this knowledge is expected to provide novel opportunities for on-farm management of soil biological resources toward enhanced disease suppression. Furthermore, molecular-based tools may enable the rapid identification of soils suppressive to specific diseases. These soils will represent important natural resources enabling the transmission of disease suppression from one soil to another by deliberate soil inoculation (94).

Functional properties of the soil microbial ecosystem are being inferred based on the taxa present. Using phylogenetic marker gene information (e.g., 16S rRNA or ITS gene sequences), bioinformatic tools such as PICRUSt [Phylogenetic Investigation of Communities by Reconstruction of Unobserved States; (110)] and FUNGuild (111) can predict the metagenome level functional content (e.g., C and N cycling genes) or functional guilds (pathogens, saprotrophs, symbionts, etc.) to provide deeper ecological insights into the functional ecology of the data sets. The expansion of these and similar tools, alongside better reference data (annotated genomes) which these tools reference, will provide further cost-effective approaches to describe the ecology and functioning of complex ecosystems such as soils.

The transfer of microbial species from one soil to another can confer new ecosystem phenotypes. This has been well-established for various microbial species such as mycorrhizal fungi, plant pathogens, and rhizobia. The movement of these taxa among soils has direct impact on the productive capacity and success of various plant species in the receiving environment (112, 113). This demonstrates the potential to manage soil biology for specific production-based and/or environmental outcomes.

Soil biology is the “engine room” that recycles plant material, either from direct inputs (leaf fall, root senescence), or secondary deposition (animal manure, urine) (114). The nutrients in these materials are either recycled within the biosphere, or mineralized into the geochemical matrix of the soil (114). In terrestrial systems, the soil microbiology provides an interface between the biological and abiotic worlds, affecting movement of essential major and minor elements between the geologic reserves and the biosphere. As such, there are a broad range of opportunities to harness the potential of soil ecosystems to optimize nutrient cycling. These include increasing the supply of many major and minor essential elements for plant use, stimulating the long-term storage of carbon in soils and promoting “closed” nutrient cycling within specific environments such that N within NO_3_ and N_2_O, for example, stay on-farm. Given our current lack of understanding of soil biology, we still have only a rudimentary knowledge of the extent of species interactions that may potentially affect critical rate regulating biogeochemical transformations in the mineralization, immobilization, and cycling of nutrients and the coupling of nutrient cycles. Indeed, it is highly likely that cryptic species and/or functional processes will have hitherto unrecognized importance in many aspects of soil nutrient cycling.

## Case Study 2: Pasture Microbiome

Grassland composition and forage production is finely balanced under the influence of interactions among many factors (115). These include the physical environment of soil, water, nutrient availability, temperature, extreme climatic events [e.g., (116)], pasture management of the grazing process (117), plant genetics (118), and the soil and plant microbiomes (see case study 1, above). Pasture-based livestock industries are primarily based on relatively simple mixtures of temperate grass and legume species as the main feed source for ruminant animals. Yet even these “simple” vegetation communities vary greatly in space and time (119), often for reasons that are not obvious using traditional scientific monitoring or analytical methods.

Well-studied components of the microbiome in grass-legume pastures involve the symbiotic association of *Epichloë* endophytes in grasses and *Rhizobium* nodules on legume roots. We illustrate ecological interactions involving these critically important microbiome components with an example based upon pasture dynamics under dairy cattle grazing in a warm-temperate region of New Zealand.

New knowledge of the ecology of pasture communities in northern New Zealand has revealed a clear instance where the microbiome drives change in community structure, with consequent feedback loops that engage other microbial communities. Sustained high densities of the root-feeding insect pest black beetle (*Heteronychus arator*) since 2007/08 in the Waikato and Bay of Plenty regions of New Zealand (120), combined with other stress factors, particularly increasing summer-autumn soil moisture deficit (121), has led to widespread but spatially disaggregated instances of near-complete failure of pastures based on perennial ryegrass (*Lolium perenne*). For example, when a ryegrass population contains a strain of the endophyte *Epichloë festucae* var. *lolii* [formerly *Neotyphodium lolii* (122)] that offers minimal protection against the insect pest black beetle, pasture collapse is observed within 2 years after sowing (123). In contrast, when the ryegrass population contains an *Epichloë* endophyte strain effective against black beetle, ryegrass populations are maintained (124).

A signature of ryegrass failure is the content of white clover (*Trifolium repens*) in the pasture. When white clover is sown together with ryegrass cultivars containing the endophyte strain AR1 (which does not protect against black beetle), the white clover content of the pasture increases rapidly. In contrast, in pastures sown with perennial ryegrass containing endophytes that are effective against black beetle such as wildtype endophyte or strain AR37 (125) the ryegrass/clover balance is stable. The plant genome does not explain this different survival pattern, although there can be subtle host genotype x endophyte strain interactions that mediate the speed and scale of change (126). The outcome of clover dominance, resulting from reduced competitive pressure from the grass (127) leads to *Rhizobium* symbiosis becoming a dominant process in the community. This illustrates the connectivity between the plant and soil microbiomes—mediated through ecological processes, in this case competition. Furthermore, clover dominance changes the nutritional composition of the feed eaten by livestock, reducing total fiber content and increasing soluble protein (128), which in turn creates a change in the rumen microbiome where the microbial composition changes in response to the altered substrate. This change results in a further interaction involving the microbiome impacting on the host animal. A direct outcome of this interaction is greater ammonia release in the rumen, flowing through to increased excretion of surplus nitrogen via higher urinary nitrogen concentrations (129) and heightened risk of nitrate leaching from beneath the urine patches returned to the grazed pasture (130).

This example illustrates the influence of, and in some case control by, the microbiome on the productivity and sustainability of a pastoral ecosystem. It highlights feedback loops, precipitated by a mis-match between the plant microbiome and the environment, led to the transformation of a grass-dominant and relatively nitrogen-efficient pasture to a legume dominant pasture with a leaky nitrogen cycle. The new pasture state with strong clover content may increase total herbage accumulation (compared with a ryegrass-dominant sward) in the short term. However, if clover contribution remains in the range of 15–30% of herbage mass (131), the consequences for the long-term yield potential of the system cannot be easily predicted. For instance, the new pasture composition may accentuate patch selection if grazing animals express partial preference for clover (132, 133). The resulting bimodal frequency of pasture mass observed in cattle grazed systems [e.g., (134)] will likely reduce the total production of the system (135). Questions that then arise include: Where and how should we intervene to manipulate the microbiome in an ecological system such as this? With what purpose and consequences? What benefit can we expect, relative to the manipulation of the plant or animal genome itself, from going down this pathway?

## Case Study 3: Rumen Microbiome

By virtue of converting human-indigestible plant polymers (cellulose and hemicellulose) into edible animal protein, ruminants enable high value food production from pasture plant resources. In the New Zealand context, ruminant animals are therefore an important part of the pastoral sector and produce a wide range of food and fiber products of considerable value to the economy. The digestion of plant material is achieved via the ruminant's specialized digestive systems, consisting of a multi-chambered stomach which supports the growth and fermentation activities of a diverse array of anaerobic microbes. The main digestive processes are carried out in the first two stomach compartments, called the reticulo-rumen, where microbes colonize and degrade forage plant material. The microbes ferment the released sugars into volatile fatty acids (VFA), which are absorbed from the reticulo-rumen and used by the animal to drive growth and formation of food and fiber products. The process is regulated so that only a partial fermentation occurs, allowing the ruminant host to absorb and utilize the intermediate fermentation products for its own metabolism and growth. The ruminant also benefits from the provision of vitamins and from the microbial cells flowing further down the digestive tract.

There has been a continual drive by livestock breeders and farmers to improve the efficiency of digestion in the rumen. Studies of the rumen microbiome have focused on understanding the contribution that the microbes make to the digestion and metabolism of particular feeds, or that are involved in production traits that are selected during animal breeding. However, microbiome analyses are increasingly being used to identify new ways to manipulate microbial metabolism, to enhance digestive capacity and drive greater output of food and fiber products by the host animal, while reducing waste or detrimental end products of the fermentation that have negative impacts on digestive efficiency, rumen function or the environment. There are many previous examples of microbial manipulations in ruminants to influence digestion, including additives such as buffers, antibiotics [ionophores and non-ionophores; (136–139)], methane inhibitors (140–148), vitamins, minerals, iso-acids, enzymes, and exogenous bacteria and/or yeast (149). These additives target different processes in the rumen and have varying degrees of effectiveness, depending on the ruminant species targeted and the diet fed to the animals. Many of these additives are non-selective or have unknown modes of action, and there is a need to have a better understanding of rumen microbiome responses so that these manipulations can be more precisely tailored for delivery of the desired improvements while removing, or minimizing, any unintended consequences.

Rapid advances in DNA and RNA sequencing, and new high throughput screening technologies for proteins and metabolites, are now making a complete description of the rumen microbiome an achievable goal (150). Combined with the ability to interpret the “omics” information using new bioinformatics approaches, this is transforming our understanding of the rumen microbial ecosystem (70, 151–154) and will inevitably lead to new ways of manipulating ruminal fermentation processes. Although these technologies are relatively new, they are being used to address recurring questions about the contribution the rumen microbiome makes to the nutritional functions of the ruminant. This will allow assessment of the types of microbes that are present, how many organisms are there, their relative quantities, and their functional role. Furthermore, as a better appreciation is gained of the importance of gastrointestinal microbes to their host, new questions around their protective, immunological, and developmental benefits to the host are being posed (155–157).

An example which illustrates the interactions between the host animal and its rumen microbiome involves methane yield differences in sheep that are related to expression of genes encoding the hydrogenotrophic methane formation pathway (158). Methane is produced in the rumen by the methanogenic archaea and is released from animals via eructation, or belching, and is also respired via the breath (159). Methane is an important agricultural greenhouse gas and has a global warming potential (GWP) of 28, meaning it is 28x the GWP of CO_2_. Agricultural methane emissions contribute ~14% of all anthropogenic emissions and therefore reducing emissions from ruminant animals is an important goal globally. While the main rumen methanogens are known, the process of methane formation is not clearly linked to either the number (160–162) or a particular community structure of methanogens (163, 164). However, it is known that the concentration of methanogenic substrates (mainly hydrogen and methyl compounds such as methanol and methylamines) and the interactions between methanogens and microbes producing and consuming hydrogen in the rumen (165, 166) are important factors contributing to methane emissions. To better understand methane formation, there has been a concerted effort to accurately measure methane emissions from ruminant animals, to examine the variation in methane yield (g methane/kg dry matter intake) between animals, and to assess the effects of different diets or dietary additives on methane output. Measurements made in sheep have shown methane yields vary considerably between individual animals within flocks (167–169), by as much as 34% between the low and high methane emission phenotypes. These variations in methane yield have been linked to differences in particle retention time in the rumen (167, 170, 171) and rumen volume (172). Furthermore, the variations were found to persist under different grazing conditions and to be a heritable trait in sheep (169). The genetic basis for the methane phenotype in sheep is indicative of a key interaction between the host animal and the rumen microbiome. Because methane is produced solely by the action of methanogenic archaea, rumen methanogens must make some contribution to the methane phenotype in sheep, either directly or via changes to the microbial community in the rumen.

To examine the contribution that the microbiome makes to methane yield, sheep with high or low emission status were rumen sampled and DNA and RNA were extracted to enable both metagenome and metatranscriptome analysis of their rumen microbiomes (158).

Surprisingly, these analyses showed no differences in the relative abundance of bacteria, archaea or eukaryotes between the low and high methane yield sheep (158). Even detailed genus-level analysis of methanogens showed only slightly elevated levels of *Methanosphaera* spp. in the low methane yield sheep and slightly higher *Methanobrevibacter gottschalkii* in the high methane yield sheep, however these were not sufficient to explain the differences in animal methane yield. An analysis of abundance of genes encoding the methanogenesis pathway also showed no significant differences, which confirmed the rRNA gene analyses. However, when the metatranscriptomic data were examined, there were clear increases in transcripts of genes encoding the methane metabolism pathway in high methane yield sheep. In particular, the genes encoding the hydrogenotrophic methanogenesis pathway (in which methane is formed from hydrogen and carbon dioxide) were significantly up-regulated compared to the methylotrophic methanogenesis pathway (where methane is formed from methyl compounds). Specifically, high methane yield sheep had high transcript levels of the methyl coenzyme M reductase enzyme (*mcr*, EC: 2.8.4.1) which catalyzes the final step in the methane formation pathway. A detailed comparison of these *mcr* genes found that they clustered into three distinct groups, called sheep rumen MCR groups 1, 2, and 3. The SRMR1 group of *mcr* genes were derived from a new group of rumen methanogens which belong to the order Methanomassiliicoccales. The SRMR2 group was identified as encoding an isozyme of methyl coenzyme M reductase (MCRII encoded by the *mrt* gene) and was found in both *Methanobrevibacter* spp. and *Methanosphaera* spp., while the SRMR3 was derived from *Methanobrevibacter* spp. only. The vast majority of methyl coenzyme M reductase transcripts were from the SRMR1 and SRMR3 groups and were 2.84- and 2.85-fold more abundant in high methane yield sheep, respectively, while SRMR2 transcripts were very low (158). These results showed that transcriptional up-regulation of the hydrogenotrophic methanogenesis pathway was an important microbial mechanism contributing to higher methane yield in sheep.

**Figure 1:**
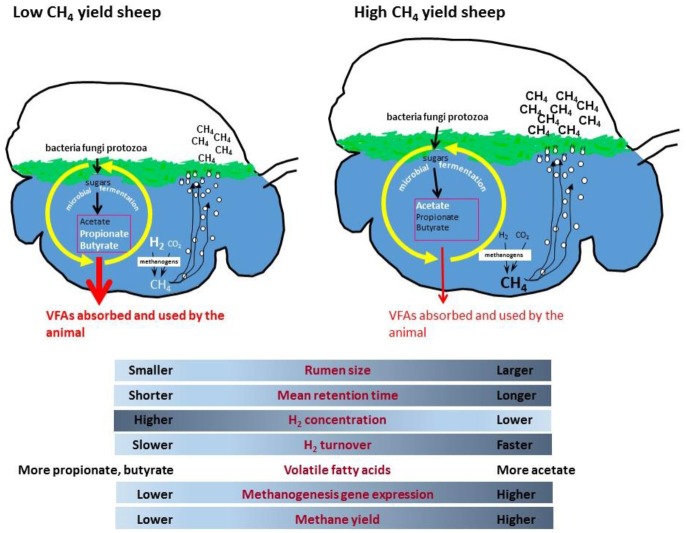
Proposed rumen model for methane yield phenotypes in sheep.

It makes biological sense that an up-regulation of methanogenesis genes in rumen methanogens results in more methane emissions from animals, but why does this happen in some sheep and not in other grazing animals? A possible mechanism has been proposed which incorporates differences in rumen size and feed particle retention time, leading to altered microbial growth kinetics and fermentation thermodynamics which affects ruminal dissolved hydrogen levels (165). It is proposed that low methane yield sheep have a smaller sized rumen, which causes increased particle passage rate that leads to higher rumen hydrogen concentrations (Figure 1). The higher hydrogen concentration causes a negative feedback that results in less hydrogen formation by fermentative microbes, leading to less methane formation. Conversely, high methane yield animals are predicted to have a larger rumen with slower particle passage, which results in lower hydrogen concentrations, enhanced hydrogen formation during fermentation, and more methane. Under ruminal conditions of slower particle passage rate and lower hydrogen concentrations, it is predicted that there is a higher turnover rate of a smaller hydrogen pool through the methanogenesis pathway to account for the elevated methane formed. The lower ruminal hydrogen concentration means that methanogens have to increase expression of methanogenesis genes to produce more enzymes to scavenge the hydrogen and maintain its turnover rate. This is because enzyme concentrations as well as substrate concentrations can limit the flux through a pathway, and increasing enzyme expression partially overcomes the limitation of lower substrate concentrations. Conversely, a high particle passage rate and high hydrogen conditions would require a lower level of expression of methanogenesis pathway genes to permit the same flux. More recent studies have shown that the dissolved hydrogen concentrations in the rumen are higher than predicted from calculations that assume equilibrium with the gas phase (173). The concentrations of hydrogen measured *in vivo* show that hydrogen is super-saturated in the rumen, and significantly affects the calculated ΔG of hydrogen-forming and hydrogen consuming reactions in the liquid phase of rumen (174).

The strong relationship between expression levels of the hydrogenotrophic methanogenesis pathways in rumen methanogens and methane yield in sheep, is the first example of rumen microbial gene expression being directly linked to an animal phenotype of relevance to environmental sustainability and production.

## Discussion

The case studies described above illustrate the complex nature of interactions within the microbiomes of the soil, plants and the rumen of grazing animals. The potential for further higher-level interdependencies and interactions between the microbiomes along the soil, plant and animal continuum dramatically increases the overall complexity in a wider ecological context. Associations between the microbiomes of soil, plants, above and below-ground animals, and the environment are massively complex. These involve soil “genotype” x plant genotype x animal genotype x rumen “genotype” × environment interactions. Thus, while there are many examples of the importance of management of individual and simple microbiomes, most ecosystem outcomes are supported by the activities of multiple consortia of microorganisms. These outcomes are the result of many microbial species and strains, with a collective functional capacity resulting in an altered ecosystem phenotype. The opportunities to harness these interactions are immense, and offer great potential if they can be understood, directed and actively managed.

## Soil-Plant Microbiome Interactions and Opportunities

Critically, the soil microbiome has a number of direct influences on plant performance. With the exception of seed-borne (vertically transmitted) endophytes, the soil biology provides the primary reservoir of microorganisms that colonize the root rhizoplane, rhizosphere, and ultimately the wider endophytic microbiome within the plant (175–179). The discovery of plant endophytes remains in its infancy, and estimates of 1 million endophytic species of higher plants may be reasonable (180). The consequences of the endophytic colonization of plants are profound, as the plant microbiome has wide ranging impacts on expression of plant phenotypes. Plant-associated microbiomes have been shown to confer drought tolerance (181), alter flowering phenology and timing (182, 183), influence plant shoot dry matter production (179), and induce systemic resistance to diseases (184). The interaction between the microbiome and plant genetics also affects aspects of plant quality via altering changes in the production of plant metabolites, or providing additional metabolic capacity via ancillary metabolic pathways encoded in the microbiome (185). An interesting example of interactions within the soil-plant microbiomes involves assessment of transgenic potato plants expressing an antimicrobial protein that is secreted into the apoplastic space between cells (186). While minor differences in the microbiomes were found in the rhizosphere of transgenic vs. non-transgenic plants, these changes were negligible compared to differences between non-transgenic plants of different potato cultivars. Another example is in flavor development in strawberries where the quantity and profile of their flavor is influenced by microbiome regulation of fuaranol synthesis (187, 188). Furthermore, the soil microbiome influences the microbial community on the grape berry and subsequent wine properties (189). It is also likely that microbiomes affect the quality of resins, fruit, honey, and essential oils (185). While the manipulation of plant traits through the microbiome have been vastly under-studied, this represents major opportunities for production of novel products or additional value of current products (190). Indeed, the microbiome background in which plants are grown can be seen to contribute to the wider *terroir* of the plant product, and may be used to add value to the provenance of products grown in different soils.

There are many direct and indirect interactions between below and above ground ecosystems, and these converge into terrestrial ecosystem function (191, 192). Collectively these express as an “ecotype,” or “functional status” to the soil. Across a multitude of functions, a “normal operating range” of soil ecosystems can be defined. These can be assessed by sampling across a range of sites to give a generalized understanding of the performance of a soil, allowing an assessment of measured vs. expected system function (81). This framework can be expanded to investigation of factors such as expression of plant-genotype effects, impacts on soil ecosystems due to disturbance (e.g., by humans or climate), and assessing ecosystem recovery. Future decisions about plant or cultivar selection for different farming systems (e.g., pastoral, arable, horticulture, and forestry) will include an understanding of the underlying soil biology. Furthermore, these decisions are likely to extend to precision use of fertilizers, agri-chemicals, and seed dressings (including biological ingredients), that consider the wider ecosystem parameters. These opportunities will need to find a balance between optimal output of products and sustainable environmental outcomes, which are not obtainable with the current laissez-faire approach.

## What Connections Exist Between Soil, Plant, and Animal Microbiomes?

**Figure 2:**
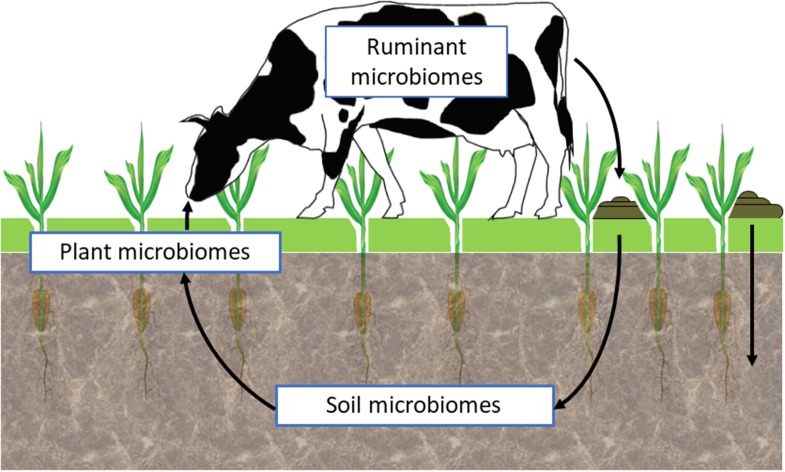
Harnessing microbiome function.

Research is now striving to understand the interactions between the soil, plant, and animal microbiomes within different environmental situations (Figure 2). Such holistic, community-level approaches to assess complex, multi-trophic linkages and communication among microorganisms, plants, and animals, within a wide environmental spatiotemporal heterogeneity, will require application of a range of emerging tools and approaches such as those based on ecological genomics (71). These will need to deliberately embrace the inherent complexity of microbiomes as “meta-ecosystem” containing an assortment of biological elements (species, mobile genetic elements), with different functional potentials resulting in an overall ecosystem phenotype. An integrative ecological genomics approach, that explores interactions among and across these meta-ecosystems and their collective ecological control, will be required to translate the biology to useful applications in a control-analysis approach (81).

As indicated in the dairy pasture dynamics case study, animal production systems in New Zealand are based on year-round grazing of ruminants on pastures, which are dominated by perennial ryegrass-clover mixtures (125). There are clear connections between the plant and animal microbiomes via the ingestion and fermentation of plant material in the rumen. However, there is very little information on how the above ground plant microbiomes (i.e., endophytic and ectophytic microbes colonizing the internal and external parts of plant stems and leaves) affect digestive processes in the rumen. It is known that as soon as plant material enters the rumen, it is colonized by a succession of different rumen microbes which initiate digestion (193–195). There is also evidence that ingested plant material continues to metabolize and undergoes a cell death response which leads to DNA fragmentation and protein breakdown, independent of rumen bacterial activities (196). This autolytic plant protein breakdown contributes to the inefficient use of plant protein which can result in much of the ingested nitrogen being lost from the animal in the form of ammonia and urea, which can cause environmental problems when excreted from the animal. The types of endo- and ecto-phytic plant microbes entering the rumen, and their activities during the plant digestion process, are poorly understood. Knowing what type of microbes are carried into the rumen in, or on, plant forages may reveal opportunities for microbial manipulation of the plant autolytic processes, allowing for the enhancement of rumen microbial colonization of the plant material or improvement of the ruminal digestion process itself.

Investigation of plant microbiomes entering the rumen may also offer some new insights and perspectives into facial eczema (FE), a significant disease in ruminants caused by saprophytic fungi growing on the dead litter at the base of pastures. The fungus, *Pithomyces chartarum*, proliferates under the warm moist conditions typically found in late summer and early autumn, and produces large numbers of spores which are subsequently ingested by the animal. In the rumen, the spores release a mycotoxin, sporidesmin, which is absorbed and causes damage to the liver and bile ducts. The damaged liver is unable to breakdown chlorophyll normally and a toxin, phylloerythrin, builds up in the blood causing sensitivity to sunlight and skin inflammation, leading to the FE symptoms of skin irritation and peeling, lowered production and sometimes death of the animal. Current management practices for FE include treatment of stock with zinc sulfate (supplied via their drinking water, drenched as a liquid product or delivered via an intraruminal slow release capsule), or applying fungicide to pastures before the spore counts become too high. There are also animal breeding programmes underway to select for FE-resistant sheep and cattle. Non-toxigenic isolates of *P. chartarum* have also been investigated as potential competitive exclusion biocontrol agents under New Zealand conditions (197). While sporidesmin levels were reduced by the application of the non-toxigenic isolates to pastures, the percentage of such isolates declined from 90 to 54% of all *P. chartarum* isolates retrieved during the 19-week trial, and after 15 months represented only 4% of the isolates from treated plots. This indicates that non-sporidesmin-producing isolates did not persist in the environment, at least under the field conditions examined. These observations suggest that better understanding of the microbiomes associated with decaying plant material in pastures, and of the ingestion and subsequent degradation of spores in the animal gut, may lead to easier and more effective means of controlling this serious animal disease.

While the interactions between plant and ruminant microbiomes are obvious, it is less appreciated that ruminants also ingest significant quantities of soil. The amounts ingested by ruminants depends on the amount of soil that becomes attached to the portions of the plant grazed by the animal, which is influenced by the weather conditions and soil type. Soil ingestion is lowest in the summer months on soils with strong structure and is highest in the winter months on soils with poor structure, where there is greater opportunity for rainfall to mediate soil transfer to the above ground parts of the forage plants (198, 199). Soil ingestion was originally studied in relation to teeth wear in sheep, as particularly abrasive soil types contribute significantly to teeth wear, limiting the productive lifetime of ewes. These studies showed that ewes can ingest up to 400 g of soil per day (198), while similar studies carried out in dairy cows found they could ingest more than 2 kg of soil per day under certain conditions (199). Soil ingestion is also a major route of uptake of trace elements for ruminants (198). The best known example of this is the uptake of cobalt (Co) to alleviate the symptoms of “bush sickness” or “wasting disease.” Co is required in ruminant diets for the bacterial synthesis in the rumen of cobalamin, which is also known as vitamin B_12_. Cobalamin is an essential cofactor for the enzyme methylmalonyl-CoA mutase, involved in an important metabolic pathway of ruminants, converting propionic acid (one of the main VFA produced by the rumen fermentation) to glucose (200, 201).

Given that soil can contain 10^9^-10^10^ organisms per g, the amount of soil ingested by ruminants reported above (198, 199) represents up to 4 ×10^11^ to 4 ×10^12^ soil microorganisms ingested by sheep per day, or 2 × 10^12^-2 × 10^13^ microorganisms per day by cattle. The potential impact of this ingested soil microbiome is estimated to be from ~8 to ~2.6% of the rumen microbiome of sheep and cattle, respectively. These estimates of soil-borne microbes entering the rumen are consistent with recent findings of a global rumen census, in which exogenous microbes (likely to be derived from ingested plant material, water or soil) on average made up ~3% of the rumen microbiome sequences (151). The changes in dairy pasture dominance by clover over ryegrass in Case Study 2 described above, has shown the indirect effects of soil microbes on ruminant production via altered forage species mix and elevated N fixation. However, there has been no examination of the direct influence that ingested soil microbes themselves might have in the rumen. The rumen and the soil environments have very different physico-chemical conditions which select for dissimilar microbiomes, therefore one would not expect soil organisms to survive, or be metabolically active, for long periods in the rumen. However, the effects of enhanced trace element supply to ruminants via ingestion of soils and the subsequent co-factor biosynthesis by bacteria in the rumen, suggest that some interactions of relevance to ruminant growth and health do occur at this interface, and are worthy of further characterization.

After digestion in the rumen, the remaining material is further fermented in the hind gut before being passed from the animal as dung. In the New Zealand dairy grazing system, dung is mainly deposited as “cow pats” onto the pasture, and eventually is broken down by a combination of microbial, insect and earthworm activity and incorporated into the soil or is volatized as ammonia. The amounts of manure produced by cattle varies considerably; beef cattle consuming 12 kg dry matter intake (DMI) per day produce about 5–6% of their body weight as manure each day (average ~27 kg wet weight), while dairy cattle with a 22 kg DMI produce closer to 70 kg per day. The microbial density in manure is roughly equivalent to the density in the rumen, but the phylogenetic distribution of microbes within the manure differs. While the rumen microbiome is usually dominated by Firmicutes and Bacteroidetes, manure can have altered Firmicutes to Bacteroidetes ratios (202) or elevated levels of Proteobacteria (203). The type of diet consumed by the animal also influences the composition of the fecal microbiome (204). Studies on the effects of manure deposited onto pastures of upland soils, indicate that dung deposition provides additional substrate for microbial growth and metabolism, and alters nutrient availability (205–207). The contribution of ruminant gut microbes in the manure, to these soil processes remains unknown, and represents a potential point of intervention to affect beneficial changes to the availability of nutrients from the soil.

## Downstream Implications for Environmental and Food Monitoring

While focusing on increasing the benefits of enhancing agricultural production through the microbiomes along the soil-plant-animal continuum, there is also a need to consider the potential effects of animal pathogens on human health. We know that changes in animal diets and/or farm systems can affect the zoonoses carried by farm animals (208–210). These zoonoses can impact on human health via multiple pathways. The first is direct animal contact which impacts predominantly on the people who work in the industry, as well as non-occupational contact (211). The second pathway is via contamination of the food products consumed (212, 213). The third major pathway is via water contamination (214) which in itself can exhibit three separate pathways such as drinking water (215), contact recreation (216) and irrigation of food crops (217). These outbreaks of zoonotic disease events can have considerable economic cost to the agricultural industries (218–220).

Genomic technologies present a major opportunity to have a transformational impact on environmental monitoring and food monitoring. This will result in increased use and adoption of genomics tools for diagnostic purposes associated with the monitoring of “risk microbes” involved with environmental health, food safety and well-being of people. Genomic techniques have already shown great potential in linking and understanding sources of food or water contamination (221–223). Highly sensitive targeted amplicon sequencing can readily detect specific pathogens and environmental metagenomics will generate huge data sets in which risk microbes can be identified.

Genomic technologies are extremely sensitive, therefore interpreting a positive signal for the presence of a DNA sequence in a sample becomes absolutely critical. There is an urgent need to establish “genomic thresholds” for water quality or food contamination to allow appropriate interpretation of genomic diagnostic data by environmental and food regulatory authorities. Similar issues are also posed for the implementation of genomic diagnostics in biosecurity decisions at border controls or clinician/veterinarian interpretations in human/animal health. A critical requirement will be the up-skilling of end-users in genomics to ensure that genomic-based diagnostic data can be effectively interpreted and appropriate actions implemented by stakeholders when enforcing policy decisions.

## Future Opportunities for Understanding the Microbiome Interactions

The principal advantage of using genomic tools to improve the understanding of microbiome interactions is the greater precision in identification and quantification of the structure of the microbial communities. Enhanced detection and characterization of the microbial members of each microbiome (the number of different species, the number of individuals within the species, and the detection of unculturable microbes), along with predictions of their metabolic capabilities from retrieved genomic information, will greatly enhance our understanding of microbiome community structure and function.

Assessment of species rank-abundance curves (RAC's) show that the soil microbiome contains many rare species (224, 225). This has particularly been brought to light with NGS-based community sequencing analysis; with increasing depth of sequencing, generally using SSU rRNA phylogeny, more species are discovered. That is, the tails of the RAC's generated for soil microbial ecosystems are very long. However, do these rare species matter in relation to soil-provided ecosystem function? In many cases, the rare biosphere is the reservoir of many novel lineages, colloquially referred to as “microbial dark matter” (226). Our understanding of these taxa is slight, particularly as many of these novel, and sometimes candidate phyla remain to be isolated in pure culture (227). As such, the ecological importance of the rare biosphere is unclear. Genomic analysis has shown that these taxa harbor unexpected metabolic features (226), and are therefore a potential source of novel enzymes and “stored ecosystem potential” (228). Furthermore, the recruitment of taxa, with unique ecophysiological adaptations, has been shown to be essential in recovery of soil ecosystem function after disturbance events, such as ammonia oxidation (229). Thus, the rare biosphere has wider impacts on ecosystem function than the total size of the community. It represents an important “seed bank” of organisms with which we may begin to have a functional role as opportunities arise, for example recruitment by a host plant or animal, or edaphic or environmental changes.

Numerous microbiome studies have been performed using rRNA gene targeted approaches. While this marker gene has worked well for many examples it is dependent on organisms within the sample having matches to the primer sequences used. New primer-independent, metagenomic shotgun sequencing approaches are rapidly increasing the volume of sequence data of microbiome samples across numerous environments along the soil-plant-grazing animal continuum. This is producing large databases of sequence information which is providing the science community with a significant resource for data mining to better understand these microbiomes, and will also act as a reference for characterization of future microbiomes. The continual increase in sequencing capacity at lower cost and access to constantly improving computational resources (e.g., more powerful data processing hardware and purpose-written, open source software) will allow new science questions to be asked about microbial functions in systems that were previously not possible. These advances will substantially improve the degree of replication and depth of sequencing required to cover the variables present in a given microbiome, or compensate for variation within samples that was not previously feasible (230).

The metagenomic shotgun sequencing of significant components of entire microbial communities is now becoming achievable at a reasonable cost. Coupled with improved computational power and bioinformatic analyses, this will dramatically improve investigation of microbiomes. Having the technologies to understand how the organisms within a microbiome interact to support ecosystem functions, such as nutrient cycling, is an exciting prospect and will undoubtedly lead to opportunities for discovery of novel microbiome features to improve ecosystem production and environmental outcomes. However, there are still significant hurdles to overcome (231, 232), and achieving a more comprehensive description of the soil-plant-grazing animal microbiome continuum would represent a remarkable advance in our ability to characterize and understand complex ecosystems.

## Future Questions for Understanding the Soil-Plant-Grazing Animal Microbiome Continuum

Understanding of the various microbiomes that make up the soil-plant-grazing animal microbiome continuum involves a major effort. Integrating metagenomic data from multiple microbiomes to get a more holistic view of ecosystems is rare, but is beginning to be addressed. Trying to understand the soil-plant-grazing animal microbiome continuum requires a clear framework, informed by the answers to some key questions. These include: how do the different microbes within a microbiome contribute to the overall phenotype? how do the different microbiomes interact with each other? and how do we move to an ecosystem-wide approach to understand the (role of the) microbiomes across the ecosystem? While these questions are answered, the main metabolic pathways in each microbiome need to be characterized along with how the composition of the microbiome can predict the phenotype. Genomics has already allowed for a vast amount of data to be generated, but the knowledge on how to translate this genomic knowledge into a phenotype requires further attention.

With the ever increasing volume of data available for each microbiome sample, microorganisms present in minute quantities will be increasingly detected with greater accuracy. This will provide a greater understanding of the relationship between the quantity of microorganisms in a sample and the contribution (“quality”) or ecological “phenotype” of that organism. For example, do microorganisms present in high frequency (quantity) contribute more to the overall phenotype than microorganisms which are rare (quality)?

Improved understanding of the microbiome composition with respect to quantity and quality will raise potential options to manipulate the microbiome to our advantage. Examples include the potential of keeping nutrients in the soil through using diverse plant genotypes/ plant species to manipulate the microbiome. The challenge ahead is to use the expanding genomics knowledge not only to increase the resilience of pastoral systems (and pasture persistence) by manipulating the microbiome, but to achieve this with less environmental impact while maintaining or improving agricultural outputs.

## Author Contributions

All authors have contributed to the writing of this manuscript and read and approved the manuscript.

### Conflict of Interest Statement

The authors declare that the research was conducted in the absence of any commercial or financial relationships that could be construed as a potential conflict of interest.
